# Mechanism of desuccinylation of G6PD mediated by SIRT7 to promote vitiligo disease progression

**DOI:** 10.1002/iid3.1341

**Published:** 2024-08-02

**Authors:** Yiyun Yang, Haidong Long, Lan Long, Bin Guo

**Affiliations:** ^1^ Department of Dermatology Longgang District Maternity & Child Healthcare Hospital of Shenzhen City (Longgang Maternity and Child Institute of Shantou University Medical College) Shenzhen China

**Keywords:** desuccinylation, G6PD, pigmentation, SIRT7, vitiligo

## Abstract

**Background:**

Sirtuin 7 (SIRT7) is pivotal in diverse diseases progression. Importantly, SIRT7 is associated with melanin production. However, whether SIRT7 regulates vitiligo is unclear. Therefore, we aimed to investigate the effects of SIRT7 on pigmentation and the modification of glucose 6‐phosphate dehydrogenase (G6PD).

**Methods:**

After knockdown SIRT7 and G6PD, pigmentation of melanocytes was evaluated using commercial kits, immunofluorescence, and Western blot analysis. The succinylation of G6PD mediated by SIRT7 was analyzed using co‐immunoprecipitation, immunofluorescence, Western blot analysis, and cycloheximide‐chase experiment.

**Results:**

We found that SIRT7 was highly expressed in vitiligo skin lesions. Knockdown of SIRT7 increased tyrosinase activity, melanin content, and the levels of α‐melanocyte‐stimulating hormone, MITF, TYR, TRP1, and TRP2. Additionally, SIRT7 directly interacted with G6PD. Silenced SIRT7 promoted the succinylation of G6PD and enhanced its protein stability. G6PD knockdown reversed the effect of reduced SIRT7 expression on melanin production.

**Conclsuion:**

Silencing of SIRT7 promotes pigmentation of melanocytes by succinylating G6PD, suggesting that SIRT7‐mediated G6PD desuccinylation may promote vitiligo progression.

## Graphical abstract

Sirtuin 7 (SIRT7) is upregulated in vitiligo lesions.

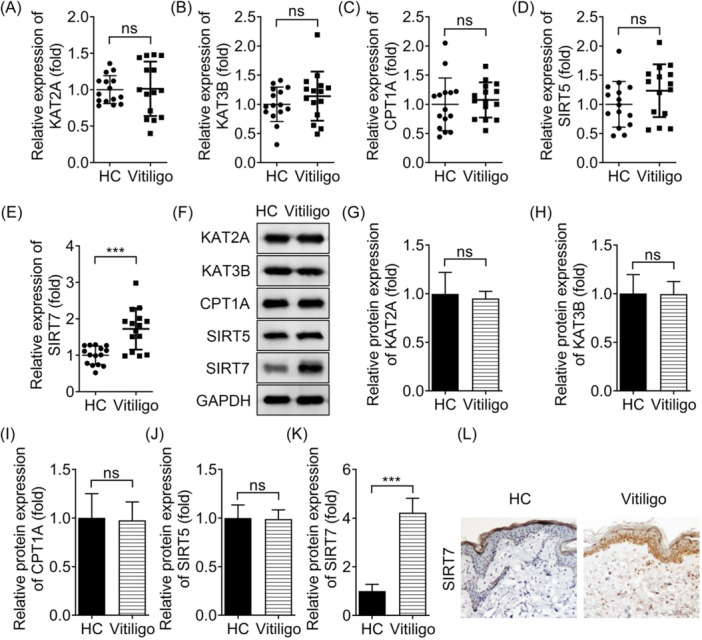

## 1 INTRODUCTION

Vitiligo is a depigmentation disorder of the skin or mucosa caused by genetic and environmental factors, which triggers an autoimmune response leading to the destruction of melanocytes.^1^, ^2^ The heritability of vitiligo is very high, genetic risk comes from common genetic variants or rare genetic variants, suggesting a polygenic, multifactorial nature of the disease.^3^ Although genetic susceptibility plays an important role in its pathogenesis, environmental factors may be even more influential as evidenced by the fact that monozygotic twins only exhibit a 23% concordance rate of the genetic susceptibility genes.^4^, ^5^ In recent years, the pathogenesis of vitiligo is well understood. It is essentially an autoimmune disease. Abnormalities of melanocytes initiate immunity, leading to adaptive immune responses and melanocyte destruction.^6^ Autoreactive cytotoxic CD8^+^ T cells produce IFN‐γ, which leading to the secretion of chemokines to accelerate the progression of vitiligo.^7^, ^8^ Therefore, the combination of environmental, genetic susceptibility, and immune theories may better explain the pathophysiology of vitiligo.

Epigenetic phenomena is heritable alternations in gene expression and functions without altering the DNA sequence, including DNA methylation, modifications of proteins, and RNA mechanisms.^9^ Histone modification is that histones undergo methylation, acetylation, ubiquitination, phosphorylation, and glycosylation mediated by related enzymes, and changes the structure of chromatin and regulates gene expression through the interaction between protein and chromatin.^10^

Lysine succinylation is a protein post‐translational modification (PTM) that regulates various pathophysiological processes. It is mediated by succinyl‐CoA cofactor, which is a metabolic intermediate during the amino acid metabolism and tricarboxylic acid (TCA) cycle. Succinylation is reversible dynamically modulated by succinyltransferase such as carnitine palmityl transferase 1A (CPT1A),^11^ lysine acetyltransferase 2A (KAT2A),^12^ and acetyltransferase 3B (KAT3B) and desuccinylases such as Sirtuin 5 (SIRT5) and Sirtuin 7 (SIRT7). Succinylation is similar to acetylation. However, compared with acetylation, succinylation greater changes the quality of substrate protein and lysine residues valence from +1 to −1, which more significantly affect protein structure and function.^13^ Increasing evidence has revealed that succinylation‐related enzymes act a crucial function in the albinism development via regulating protein succinylation.^14^ Moreover, succinylation has been reported to regulate innate immunity and inflammation.^15^ However, the succinylation in vitiligo remained unclear.

Oxidative stress event initiates the occurence of vitiligo. Glucose 6‐phosphate dehydrogenase (G6PD) is an oxidative stress defense regulator during vitiligo.^16^, ^17^ A previous study has revealed that G6PD activity and polymorphism is associated with insufficient melanocyte activity and oxidative stress response in vitiligo in the Gujarat population.^18^ Therefore, the progression of vitiligo may be due to oxidative damage mediated by impaired G6PD levels.

Here, we found that SIRT7 was highly expressed in vitiligo skin lesions, and SIRT7 promoted pigmentation of melanocytes by mediating desuccinylation of G6PD, indicating a new approach for the treatment of vitiligo.

## 2 METHODS

### 2.1 Clinical sample collection

Lesional skin tissues and adjacent non‐lesional tissues were acquired from patients with vitiligo (*n* = 15). Additionally, healthy skin was obtained from healthy subjects (*n* = 15). These pathological or normal tissues were confirmed by pathological examination. Patients with systemic disease and treatment before sample collection were excluded. Healthy volunteers had no family history of vitiligo and chronic skin diseases. Each participant provided written informed consent before specimen collection. This study was provided by the Ethics Committee of Longgang District Maternity & Child Healthcare Hospital of Shenzhen City.

### 2.2 Cell culture

Vitiligo melanocytes (PIG3V) were purchased from Biovector Science Lab, Inc. and were maintained in medium 254 (M254500; Gibco) supplemented with 5% fetal bovine serum (FBS; 10099141 C; Gibco) at 37°C with 5% CO_2_ and 95% humidity.

### 2.3 Cell transfection

Cells (1 × 10^5^ cells/plate) were inoculated in six‐well plates. Short hairpin RNA (shRNA) (sh)‐SIRT7 (5'‐GCCAAATACTTGGTCGTCTAC‐3'), sh‐G6PD (5'‐GGGCTATTTCGATGAATTTGG‐3'), and the negative control (sh‐NC; 5'‐CAACAAGATGAAGAGCACCAA‐3') were synthesized by Sangon (Shanghai, China). PIG3V cells were transfected with these shRNAs with liposome™ 3000 reagent (L3000075; Invitrogen) for 48 h. Quantitative reverse transcription polymerase chain reaction (qRT‐PCR) was used to confirm successful transfection in the follow‐up experiment.

### 2.4 qRT‐PCR

Total RNA was isolated from skins and cells using a total RNA extraction kit (R1200; Solaibio). RNA purity was determined by A260/280 ratio with 1 μL RNA sample, and the integrity was confirmed using 1% agarose gel electrophoresis using the same amount RNA. Reverse transcription was conducted using 2 μg mRNA to synthesize cDNA first chain by a cDNA synthesis kit (D7168L; Beyotime). cDNA (5 μL) was used for qPCR with the 2 × SYBR Green PCRMaster mix (SR1110; Solaibio). Reaction conditions were as follows: 95°C for 3 min, 95°C for 20 s (denaturation) and 60°C for 30 s (annealing/extension), 40 cycles. After at least 3 repetitions, the $2^{-{\mathrm{\Delta \Delta C}}_{t}}$ method was applied to calculate the results. The primer sequences used here are listed below: KAT2A sense, 5'‐TGGAGCCTGTGAAGAAGTCG‐3', antisense, 5'‐GCCGCTCAGTCATGGTCT‐3'; KAT3B, sense, 5'‐GAGCACCCGTTGGACTTG‐3', antisense, 5'‐TCGGCATCTGATTTACTTGA‐3'; CPT1A sense, 5'‐AAATTACGTGAGCGACTGG‐3', antisense, 5'‐CTGCCTGAATGTGAGTTGGA‐3'; SIRT5 sense, 5'‐AAGGCTGGCACCAAGAAC‐3', antisense, 5'‐TCCTGATAAAGCTGGACAAA‐3'; SIRT7 sense, 5'‐CAGGAGGAGGCAGCGTCTA‐3', antisense, 5'‐CTCAGGTCGGCAGCACTAA‐3'; G6PD sense, 5' CCTACGGCAACAGATACAAGA 3', antisense, 5' GCCCTCATACTGGAAACCC 3'; GAPDH sense, 5'‐GCAAGTTCAACGGCACAG‐3', antisense, 5'‐CGCCAGTAGACTCCACGAC‐3'.

### 2.5 Analysis of cell proliferation

Cells were seeded in 96‐well plates and maintained in cultured medium (100 μL) at 37°C until cell confluence reached 70‐80%. After incubating for 0, 24, 48, and 72 h, cell counting kit‐8 (CCK‐8) reagent (10 μL) was added to the plates. The 96‐well plates were placed in a microplate reader and the absorbance value was measured at 450 nm.

### 2.6 Western blot analysis

RIPA lysis buffer supplemented with benzoyl fluoride (PMSF) was used to extract proteins. Protein concentration was examined using a Pierce^TM^ bicinchoninic acid kit (23,250; Thermo Scientific) and the same amount protein (30 µg) was loaded into each lane of 10% sodium dodecyl sulfate‐polyacrylamide gel electrophoresis (SDS‐PAGE) for separation. Next, the separated proteins were transferred onto PVDF membranes and blocked using 5% nonfat milk for 1 h. Following primary antibodies incubation at 4°C for 12 h, the membranes were further incubated with secondary antibody for 1 h. After washing with tris‐buffered saline tween‐20, a Novex™ enhanced chemiluminescence substrate reagent kit (WP20005; Invitrogen) were applied to visualize protein bands.

The information of primary antibodies (Abcam) are listed below: anti‐KAT2A (ab153903, 1/1000), anti‐KAT3B (ab275378, 1/1000), anti‐CPT1A (ab234111, 1/1000), anti‐SIRT5 (ab105040, 1/1000), anti‐SIRT7 (ab259968, 1/1000), anti‐MITF (ab140606, 1/200), anti‐TYR (ab170905, 1/2000), anti‐TRP1 (ab235447, 1/1000), anti‐TRP2 (ab74073, 1/1000), anti‐G6PD (ab133525, 1/5000), anti‐succinyllysine (PTM‐401, 1/1000; PTMBIO), anti‐GAPDH (ab128915, 1/20000). HRP‐conjugated goat anti‐rabbit (ab6721, 1/3000) is the secondary antibody.

### 2.7 Immunohistochemistry (IHC)

Paraffin sections of skin were routinely dewaxed, rehydrated, and incubated with 3% hydrogen peroxide (H_2_O_2_) at 37°C for 10 min to inactivate endogenous peroxides. After washing using distilled water, the sections were boiled in citrate buffer for antigen repair. The sections were sealed with bovine serum albumin (BSA) at 37°C for 0.5 h after water and PBS rinse and incubated with primary antibody (anti‐SIRT7: ab259968, 1/100; Abcam) for 12 h at 4°C. The sections were washed with PBS and incubated with second antibody (ab6721, 1/1000, Abcam) for 0.5 h at 37°C. Finally, all sections were re‐stained with 3, 3'‐diaminobenzidine (DAB; D12384, Sigma), and images were obtained with an optical microscope.

### 2.8 Determination of melanin content and tyrosinase activity

To detect melanin content, the transfected cells were lysed in phosphate buffer (0.1 M, pH 6.8) and diluted in 1 M sodium hydroxide. To measure tyrosinase activity, cells were incubated with 2 mM levodopa (333786, Sigma) in PBS at 37°C for 90 min. After treatment, the opticaldelnsity was examined by a microplate reader at 490 nm to calculate melanin content and tyrosinase activity.

### 2.9 Immunofluorescence staining

PIG3V cell slides were fixed in 4% paraformaldehyde (158,127, Sigma), incubated with 3% H_2_O_2_ for 30 min, and permeabilized using 0.3% Triton X‐100 (HY‐Y1883A, MedChemExpress). After blocking using BSA for 10 min, primary antibodies were incubated with cell slides for 12 h at 4°C, and secondary antibodies were incubated with slides for 0.5 h. Anti‐α‐melanocyte‐stimulating hormone (α‐MSH) (ab254257, 1/100), anti‐G6PD (ab133525, 1/100), and anti‐SIRT7 (ab259968, 1/100) were used as primary antibodies. Alexa Fluor 647 goat anti‐mouse IgG (ab150115, 1/200) and Alexa Fluor 488 goat anti‐rabbit IgG (ab150077, 1/200) were used as secondary antibodies. All antibodies were acquired from Abcam. 4',6‐Diamidino‐2‐phenylindole (DAPI; D9542, Sigma) was applied to stain nucleus. Fluorescence was captured by confocal microscopy.

### 2.10 Co‐immunoprecipitation

The cells were lysed with NP‐40 lysis buffer, and centrifuged to collect the lysate (the supernatant). The supernatant was shaken with antibodies (anti‐IgG, ab172730; anti‐SIRT7, ab259968; anti‐G6PD, ab210702) at 4°C overnight. Protein A + G magnetic beads (78609, Thermo Scientific) were incubated with the samples for 1 h. The mixture was washed using lysis buffer three times, and the beads were eluted with 100 µL eluent for 10 min. SDS‐PAGE was performed after elution. Protein levels were measured using Western blot analysis.

### 2.11 Statistical analysis

All data acquired from at least three replicates were analyzed using the GraphPad Prism software (version 8), and results were expressed as mean ± SD. Data conform to normal distribution. Student's *T* test was used for analyzing the difference between two groups, and difference among multiple groups was analyzed one‐way analysis of variance followed by Tukey post hoc test. *p* < .05 was considered statistically significant.

## 3 RESULTS

### 3.1 SIRT7 is upregulated in diseased skin tissue of patients with vitiligo

**Figure 1:**
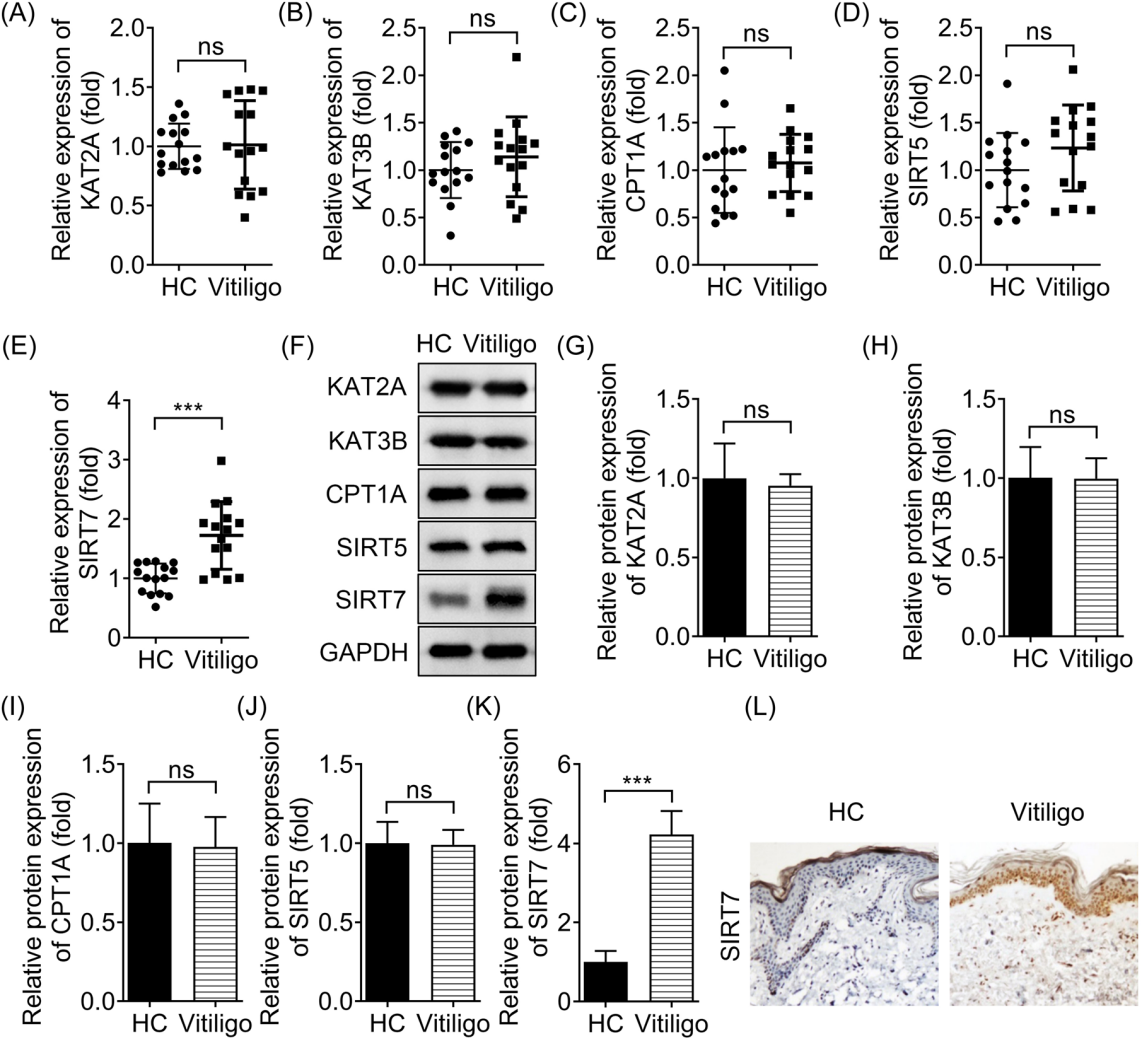
SIRT7 expression is upregulated in vitiligo skin. Vitiligo skin tissues were obtained from patients, and normal tissues were obtained from healthy control. Then, (A−E) the expressions of KAT2A, KAT3B, CPT1A, SIRT5 and SIRT7 were examined by qRT‐PCR. (F−K) The protein levels of KAT2A, KAT3B, CPT1A, SIRT5 and SIRT7 were examined using Western blot analysis and quantified. (L) SIRT7 levels in vitiligo and healthy skin tissues was detected using immunohistochemistry. ***p < .001. CPT1A, carnitine palmityl transferase 1A; KAT2A, acetyltransferase 2A; KAT3B, acetyltransferase 3B; ns, no significant; SIRT5, Sirtuin 5; SIRT7, Sirtuin 7.

The levels of KAT2A, KAT3B, CPT1A, SIRT5, and SIRT7 in skin tissues were detected by qRT‐PCR and Western blot, respectively. The results showed that only SIRT7 levels, rather than KAT2A, KAT3B, CPT1A, and SIRT5 levels, in skin with vitiligo lesions from patients were higher than that in healthy control skin (Figure 1A−F). Morover, SIRT7 levels were highly expressed in lesional skin compared with adjacent normal skin from patients with vitiligo (Supporting Information S1: Figure 1). Then, SIRT7 expression levels in skin tissues with vitiligo were detected using IHC. We found that SIRT7 expression in skin tissues with vitiligo lesions was significantly increased, compared with healthy controls (Figure 1G). Moreover, SIRT7 was mainly found in the stratum corneum of healthy skin, whereas it was mainly present in the basal layer of diseased skin (Figure 1G).

### 3.2 SIRT7 inhibits pigmentation of melanocytes

**Figure 2:**
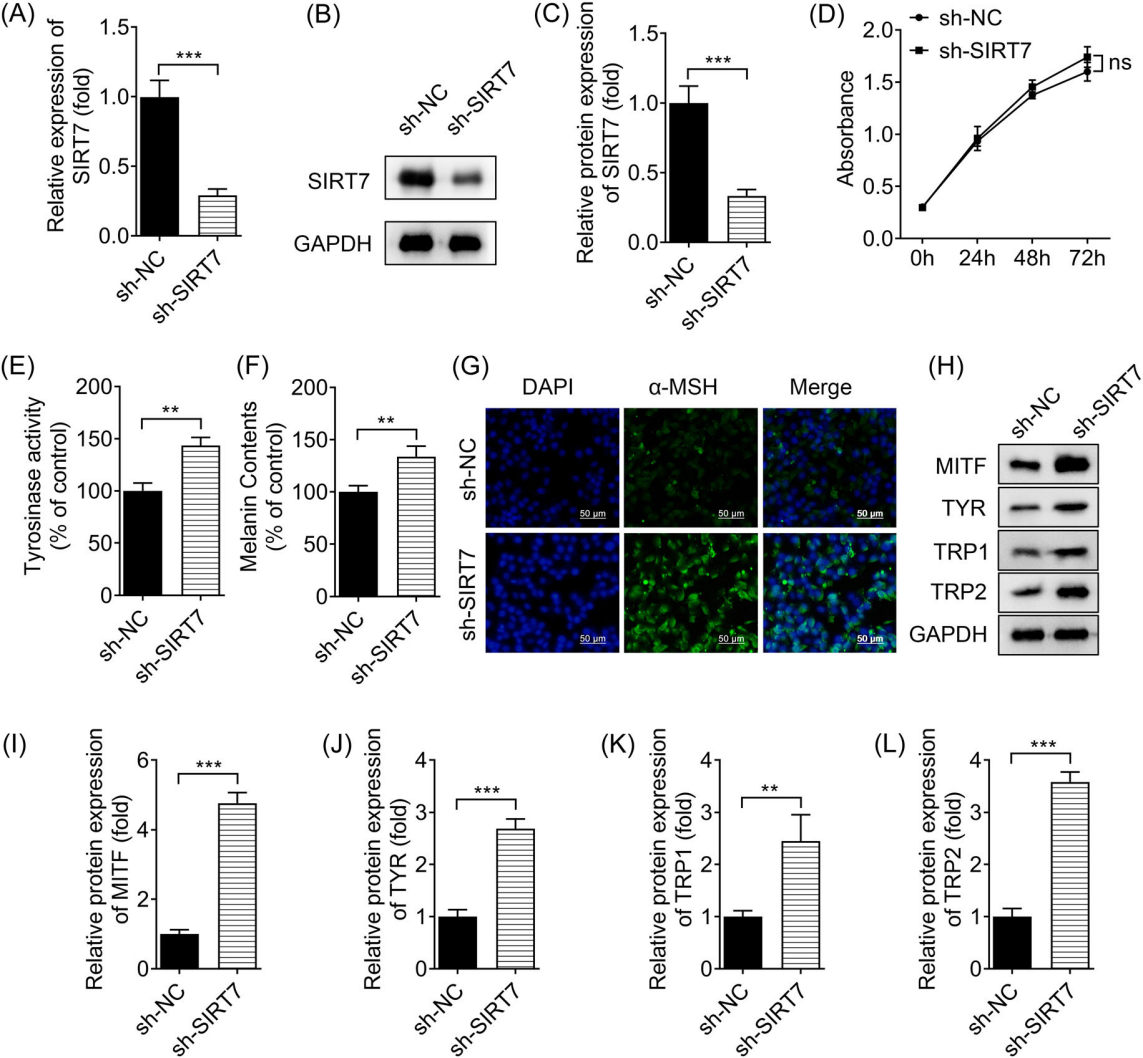
SIRT7 inhibits pigmentation of melanocytes. (A) After sh‐SIRT7 transfection, SIRT7 expression was detected by qRT‐PCR. (B) After sh‐SIRT7 transfection, SIRT7 levels were measured using Western blot analysis and (C) quantified using densitometry. (D) CCK8 assessed cell viability. (E, F) Tyrosinase activity and melanin content. (G) The expression of α‐MSH was visualized using immunofluorescence (scale bar: 50 μm). (H) The levels of melanogenation‐related proteins MITF, TYR, TRP1 and TRP2 were detected by Western blot analysis and were calculated using densitometry in (I−L). **p < .01, ***p < .001. CCK‐8, cell counting kit‐8; ns, no significant; SIRT7, Sirtuin 7.

To clarify the regulation of SIRT7 in melanin production, SIRT7 gene was knocked down (Figure 2A−C). CCK8 results showed that SIRT7 knockdown did not affect cell proliferation (Figure 2D). However, downregulation of SIRT7 significantly increased tyrosinase activity (Figure 2E). Consistent with the change in tyrosinase activity, melanin content (Figure 2F) and a‐MSH expression (Figure 2G) were significantly increased by SIRT7 silence. In addition, Western blot analysis results showed that silencing of SIRT7 increased MITF, TYR, TRP1, and TRP2 protein levels, which were linked to melanin production (Figure 2H−L).

### 3.3 SIRT7 directly targets G6PD in cells

**Figure 3:**
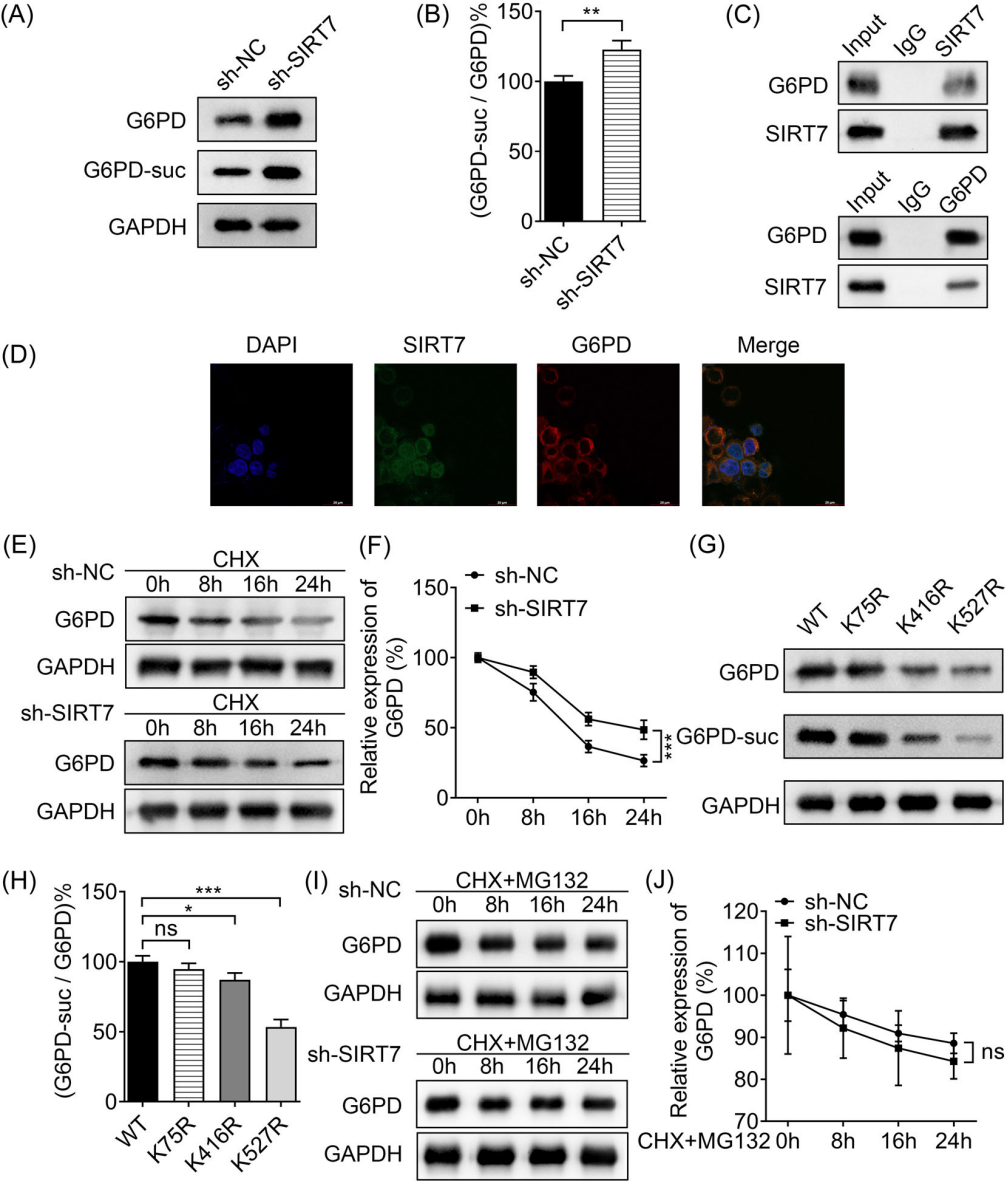
SIRT7 desuccinylates G6PD to affect protein stability. (A) Western blot analysis detected the expressions of G6PD and G6PD‐suc. (B) The percentage of G6PD‐suc/G6PD was quantified. The binding of SIRT7 and G6PD was verified by (C) co‐IP and (D) immunofluorescence (scale bar: 20 μm). (E, F) The stability of G6PD was detected by CHX‐phase experiments. (G) The levels of G6PD and G6PD‐suc when G6PD was wild‐type or mutated at K75, K416, and K527 sites. (H) G6PD‐suc/G6PD percentage was quantified. (I, J) After MG132 was added to inhibit proteasome degradation, G6PD protein stability was examined using CHX‐phase experiments. *p < .05, **p < .01, ***p < .001. CHX, cycloheximide; co‐IP, co‐immunoprecipitation; G6PD, glucose 6‐phosphate dehydrogenase; ns, no significant; SIRT7, Sirtuin 7.

To explore the modification of SIRT7 on G6PD in vitiligo, we found that silencing of SIRT7 elevated the protein and succinylation levels of G6PD in PIG3V cells, assessing by Western blot analysis (Figure 3A,B). Co‐immunoprecipitation was performed to analyze the combination of SIRT7 and G6PD, and the data revealed that SIRT7 interacted with G6PD (Figure 3C). The results of immunofluorescence further verified the binding of SIRT7 and G6PD (Figure 3D). When SIRT7 gene was knocked down, the half‐life of G6PD was increased, suggesting protein stability was enhanced (Figure 3E,F). In addition, G6PD succinylation and protein levels were decreased when G6PD K461 and K527 sites were mutated, compared with WT G6PD; however, K75 site mutation did not affect G6PD protein and succinylation levels (Figure 3G,H). After MG132 (a proteasome inhibitor) treatment, the half‐life of G6PD was failed to affected by SIRT7 (Figure 3I,J). Collectively, SIRT7 knockdown succinylates G6PD at K461 and K527 sites to enhance protein stability, then upregulating G6PD protein levels.

### 3.4 SIRT7 knockdown promotes pigmentation of melanocytes by upregulating G6PD

**Figure 4:**
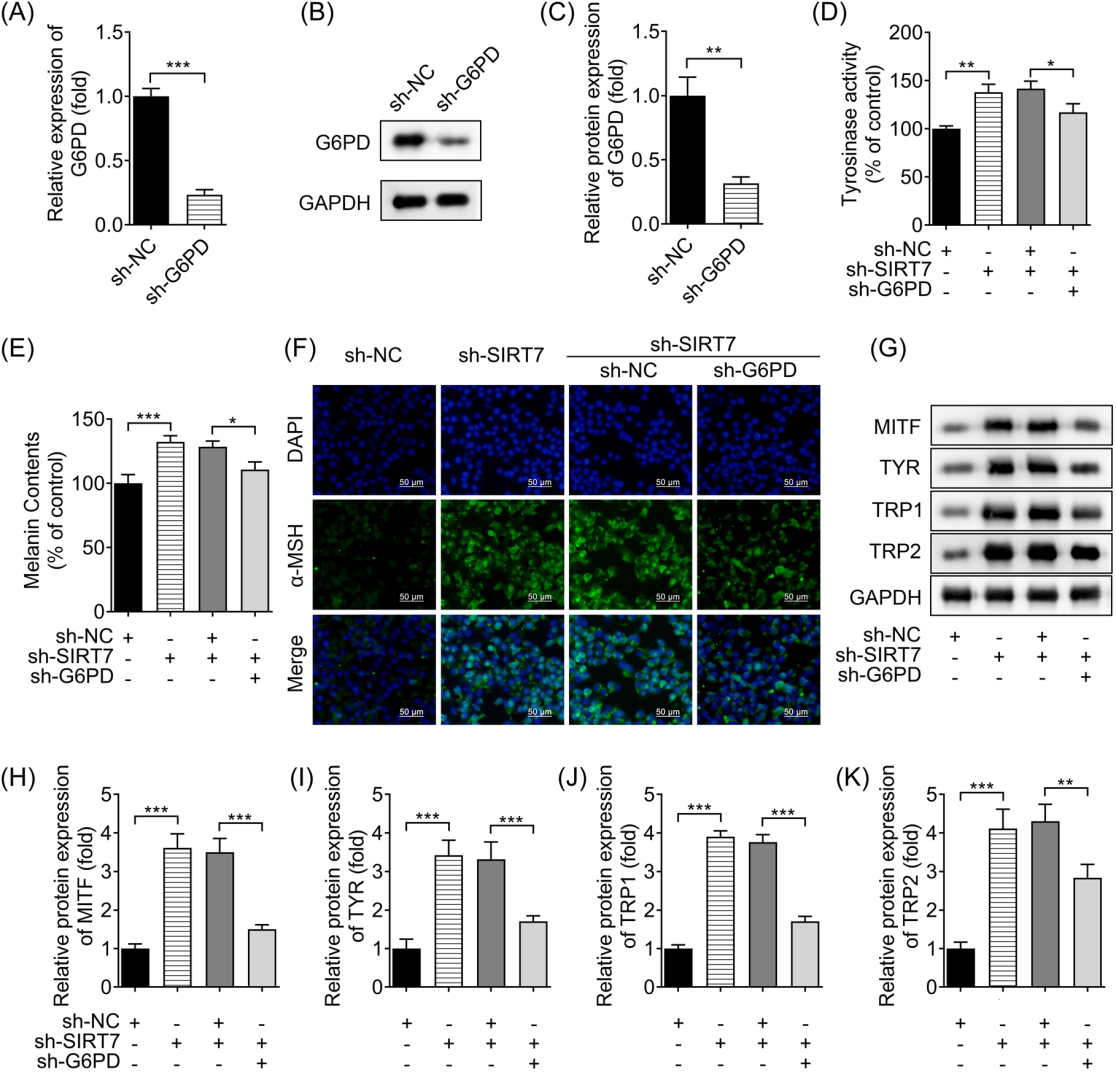
SIRT7 knockdown promotes pigmentation of melanocytes by upregulating G6PD. (A) G6PD mRNA expression and (B) protein levels after sh‐G6PD transfection were evaluated using qRT‐PCR and Western blot analysis. (C) G6PD protein levels were quantified following transfection. The (D, E) Tyrosinase activity and melanin content. (F) Immunofluorescence was applied to detect α‐MSH expression (scale bar: 50 μm). (G) Melanogenation‐related proteins MITF, TYR, TRP1 and TRP2 were detected by Western blot analysis. (H) MITF, (I) TYR, (J) TRP1 and (K) TRP2 protein levels were quantified using densitometry. *p < .05, **p < .01, ***p < .001. G6PD, glucose 6‐phosphate dehydrogenase; qRT‐PCR, quantitative reverse transcription polymerase chain reaction; SIRT7, Sirtuin 7.

Next, we identified the effect of G6PD on SIRT7‐mediated pigmentation of melanocytes using rescue experiments. G6PD expression was reduced after sh‐G6PD transfection (Figure 4A−C). Next, we found that knockdown of G6PD reversed the increase of tyrosinase activity and melanin content induced by SIRT7 silence (Figure 4D,E). Moreover, α‐MSH expression was increased after SIRT7 knockdown, and was abrogated by G6PD knockdown (Figure 4F). The levels of melanogenic‐related proteins, including MITF, TYR, TRP1, and TRP2, were increased after SIRT7 knockdown, whereas silenced G6PD abrogated this increase (Figure 4G−K). These results suggest that SIRT7 modulates G6PD to inhibit pigmentation.

## 4 DISCUSSION

Herein, we identified that SIRT7 was overexpressed in vitiligo skins. In addition, we also confirmed that SIRT7 could inhibit melanin production by increasing tyrosinase activity, melanin content and upregulating melanogenic‐related proteins levels. Subsequently, we found that SIRT7 regulated the succinylation of G6PD in melanocytes. We further demonstrated that SIRT7 promoted repigmentation of melanocytes by mediating G6PD, suggesting that SIRT7‐mediated desuccinylation of G6PD promotes the progression of vitiligo disease, and more importantly, the specific downregulation of SIRT7 might be a promising target for vitiligo therapy.

The main characterize of vitiligo is gradual decolorization caused by the destroy of epidermal melanocytes.^19^, ^20^ The pathogenesis of vitiligo is complex, and it is currently widely accepted that both genetic and environmental factors influence melanocyte function, ultimately resulting in T‐cell‐mediated immune destruction. As a member of the SIRT family, SIRT7 has been reported to affect numerous diseases, such as cancers, cardiovascular disease, inflammation, digestive system diseases, and nervous system diseases.^21^ SIRT7 is a crucial inflammation and oxidative stress modulators. SIRT7 regulates inflammation response. For example, under inflammation conditions, SIRT7 regulates TLR2 to improve reduction of immune reactivity in skin.^22^ Besides, depletion of SIRT7 inhibits LPS‐induced endothelial inflammation in acute lung injury.^23^ Moreover, SIRT7 can be activated by antioxidants, and the levels are downregulated under oxidative stress.^24^ Since oxidative stress initiates the loss of melanocytes, which is also affected by immune‐mediated inflammation, we hypothesized that SIRT7 may be involved in vitiligo progression.^25^ In the current study, we identified that SIRT7 expression was elevated in vitiligo lesions for the first time, suggesting SIRT7 regulates vitiligo progression. There is a contradiction here. Oxidative stress triggers the occurrence of vitiligo, and SIRT7 has antioxidant effects; however, we found that SIRT7 was highly expressed in vitiligo, indicating that it did not serve antioxidant function. Therefore, we speculated that there may be other factors more important than oxidative stress affecting the expression of SIRT7.

Melanin synthesis contributes to maintaining normal skin color to alleviating vitiligo.^26^, ^27^ Thus, we investigated the effect of SIRT7 on melanin production. Here, we knocked down SIRT7 in melanocytes. We found that silencing of SIRT7 increased melanin content and tyrosinase activity and meantime enhanced a‐MSH levels. As is well‐known, three members in the tyrosine gene family, including TYR, TRP1, and TRP2, are involved in this complex process of melanin synthesis.^28^ MITF participates in the whole melanin synthesis process via regulating the expressions of TYR, TRP1 and TRP2.^29^, ^30^ We found that SIRT7 knockdown elevated MITF, TYR, TRP1 and TRP2 protein levels. Taken together, SIRT7 inhibits melanin production, and may thereby accelerating vitiligo progression.

Next, since SIRT7 is a desuccinylase, we presented evidence that the SIRT7‐mediated desuccinylation of G6PD contributed to the progression of vitiligo. Silencing of SIRT7 facilitated the succinylation of G6PD and increased its protein levels by enhancing the stability. Growing evidence has emphasized that G6PD is involved in the pathogenesis of vitiligo. G6PD activity and levels are lower in patients with vitiligo than that in the control.^31^, ^32^ Moreover, G6PD play a central role in oxidative stress. It maintains cellular redox balance by resisting oxidative stress.^33^ In addition, G6PD is also associated with skin inflammation.^34^ In our study, we investigated the impact of G6PD on melanin synthesis. We clarified that silencing of G6PD impeded melanin synthesis of melanocytes caused by SIRT7 knockdown. The results suggest that SIRT7 promotes the progression of vitiligo by desuccinylating G6PD.

In summary, our findings suggest that SIRT7 suppresses pigmentation of melanocytes in vitiligo progress by facilitating desuccinylation of G6PD, contributing to the targeted therapies for vitiligo.

## AUTHOR CONTRIBUTIONS

All authors participated in the design, interpretation of the studies and analysis of the data and review of the manuscript. Yiyun Yang drafted the work and revised it critically for important intellectual content and made substantial contributions to the conception and design of the work. Haidong Long, Lan Long and Haidong Long, Lan Long were responsible for the acquisition, analysis, or interpretation of data for the work. All authors read and approved the final manuscript.

## CONFLICT OF INTEREST STATEMENT

The authors declare no conflict of interest.

## Supporting information

Supplemental Figure S1. SIRT7 expression is elevated in lesional skin tissues from patients with vitiligo. Lesional and adjacent normal skins were acquired from patients with vitiligo, (A‐E) RT‐qPCR and (F) western blot were used to measure KAT2A, KAT3B, CPT1A, SIRT5 and SIRT7 mRNA and protein levels. (G‐K) Protein levels were quantified. ***P<0.001. ns, no significant.

## Data Availability

The data sets used and/or analyzed during the current study are available from the corresponding author on reasonable request.
