# Supplementary figures and images for: Mechanism of desuccinylation of G6PD mediated by SIRT7 to promote vitiligo disease progression
Source: Immun Inflamm Dis. 2024 Aug 2;12(8):e1341. doi: 10.1002/iid3.1341 (PMC11295095; doi:10.1002/iid3.1341)

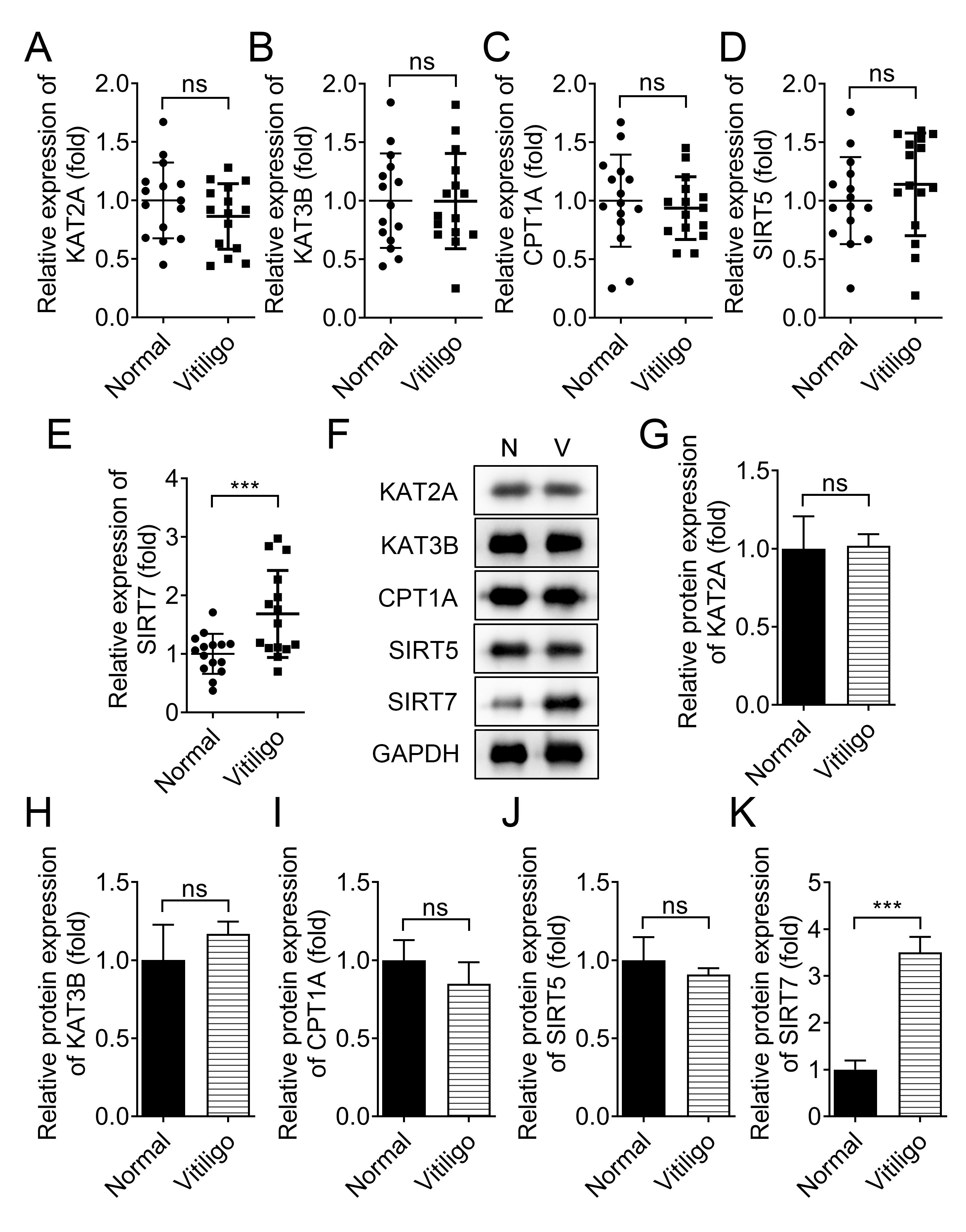

Supplement: Supplementary file 1 — Supplemental Figure S1. SIRT7 expression is elevated in lesional skin tissues from patients with vitiligo. Lesional and adjacent normal skins were acquired from patients with vitiligo, (A‐E) RT‐qPCR and (F) western blot were used to measure KAT2A, KAT3B, CPT1A, SIRT5 and SIRT7 mRNA and protein levels. (G‐K) Protein levels were quantified. ***P<0.001. ns, no significant. [file IID3-12-e1341-s001.jpg]
